# Reaching the Hard-to-Reach: A Probability Sampling Method for Assessing Prevalence of Driving under the Influence after Drinking in Alcohol Outlets

**DOI:** 10.1371/journal.pone.0034104

**Published:** 2012-04-13

**Authors:** Raquel De Boni, Pedro Luis do Nascimento Silva, Francisco Inácio Bastos, Flavio Pechansky, Mauricio Teixeira Leite de Vasconcellos

**Affiliations:** 1 Center for Drug and Alcohol Research, Federal University of Rio Grande do Sul and Hospital de Clínicas de Porto Alegre, Porto Alegre, Brazil; 2 National School of Statistics, Brazilian Geography and Statistics Institute, Rio de Janeiro, Brazil; 3 Department of Health Information, Oswaldo Cruz Foundation, Rio de Janeiro, Brazil; 4 Evandro Chagas Clinical Research Institute, Oswaldo Cruz Foundation, Rio de Janeiro, Brazil; 5 Fulbright/CAPES visiting scholar, Brown University, Rhode Island, United States of America; University of Granada, Spain

## Abstract

Drinking alcoholic beverages in places such as bars and clubs may be associated with harmful consequences such as violence and impaired driving. However, methods for obtaining probabilistic samples of drivers who drink at these places remain a challenge – since there is no a priori information on this mobile population – and must be continually improved. This paper describes the procedures adopted in the selection of a population-based sample of drivers who drank at alcohol selling outlets in Porto Alegre, Brazil, which we used to estimate the prevalence of intention to drive under the influence of alcohol. The sampling strategy comprises a stratified three-stage cluster sampling: 1) census enumeration areas (CEA) were stratified by alcohol outlets (AO) density and sampled with probability proportional to the number of AOs in each CEA; 2) combinations of outlets and shifts (COS) were stratified by prevalence of alcohol-related traffic crashes and sampled with probability proportional to their squared duration in hours; and, 3) drivers who drank at the selected COS were stratified by their intention to drive and sampled using inverse sampling. Sample weights were calibrated using a post-stratification estimator. 3,118 individuals were approached and 683 drivers interviewed, leading to an estimate that 56.3% (SE = 3,5%) of the drivers intended to drive after drinking in less than one hour after the interview. Prevalence was also estimated by sex and broad age groups. The combined use of stratification and inverse sampling enabled a good trade-off between resource and time allocation, while preserving the ability to generalize the findings. The current strategy can be viewed as a step forward in the efforts to improve surveys and estimation for hard-to-reach, mobile populations.

## Introduction

The harmful use of alcohol is the leading risk factor for death among men aged 15–59 years, and road traffic accidents rank second on global alcohol-attributable deaths [1]. In Brazil, traffic crashes (TC) caused 38,737 fatal victims in 2008 [2], and a few studies conducted in the country indicate that between 30–50% of these victims had a positive blood alcohol concentration (BAC) [3], [4]. Despite such disquieting figures, victims of TC are not systematically examined with the help of breathalyzers, rendering the report of non-fatal accidents associated with the misuse of alcohol probably underestimated. As of June 2008, a zero tolerance law was adopted in the country aiming at minimizing these figures and curbing driving under the influence of alcohol (DUI). However, much has still to be done in terms of a comprehensive enforcement of such legislation in Brazil, as has been implemented in affluent countries [1], [5], [6]. Enforcement through random breath testing and sobriety checkpoints has been erratically implemented across the different Brazilian regions and localities and remains a formidable challenge in a continental-sized and deeply heterogeneous country.

Alcohol availability remains high in Brazil [7], [8]. The government does not control alcohol sales and distribution through sanctioned licensing systems or monopolies, and any commercial establishment linked to food distribution or adult entertainment may sell alcoholic beverages at its discretion. Also, there are no restrictions in terms of the density of outlets and/or their operation (e.g. days and hours they may remain open), which could help reduce the number of accidents and related fatalities [9], [10]. Literature already indicates that consuming alcoholic beverages in places like bars and restaurants (alcohol outlets with consumption on-premises) increases the chance for DUI [11], [12], and the association of AO density and traffic crashes (TC) is under study worldwide [13]–[15]. A comprehensive assessment of this association is key, since the allocation and availability of such outlets can and should be part of public policies aiming at reducing alcohol-related harms. However, studies targeting a representative sample of people who purchase and consume alcoholic beverages on the premises and then drive are extremely rare due to the difficulties to define a valid sampling frame for this mobile population.

Most studies have used indirect estimation methods and/or are ecological, in the sense that individual-level data are usually not collected through face-to-face interviews on AO: some studies use telephone-based surveys [16], others obtain data from places where major harms associated with immoderate consumption are reported, such as emergency rooms [17], and analyses typically consider data aggregated at different levels, such as neighborhoods [18], counties [19], or states [20]. Recent studies have been using sophisticated methods, e.g. the portal survey methodology, in the assessment of alcohol and alcohol-related harms in context. Notwithstanding these improvements, the external validity of such methods remains far from optimal [21]–[23].

Different sampling methods have been used for the sake of better estimating alcohol and drug using patterns, as well as their harmful consequences. Some of these methods are based on the structure and dynamic of networks, such as the classic snowball sampling [24] and more recently respondent-driven sampling [25], [26]. The main limitation of such methods refers to the difficulty to obtain population-based estimates, due to a variety of biases and bottlenecks [27]. Another limitation is due to the fact that methods such as classic snowball or respondent-driven sampling correspond, after Valente’s typology, to “sequenced data”, i.e. networks that are situated in between egocentric or “local networks” and “complete network data”. The latter corresponds to comprehensive assessments of a given community, using either a census or a quasi-census (saturation) approach [28].

Time location/space sampling (TLS) has also been used to assess and sample hard-to-reach populations [29]–[31]. In this venue-based kind of study sampling strategies usually comprise two or three steps [32]: places are randomly selected (usually after preliminary mapping), then days and periods of time people could and should be interviewed in the different scenes, then individuals, selected with systematic sampling. Although considered as a probability-based method, the accurate and precise estimation of individuals comprising this third step is not always possible. Such limitations and caveats can be properly addressed through the use of carefully designed probability samples.

On the other hand, obtaining a strict probability sample of drivers who attend AOs is challenging. Clients can consume alcoholic beverages in different settings, hours, days of the week (including weekends), and their habits may vary in different periods of the year (e.g. seasons, rainy versus sunny days). All such factors make this a mobile, dynamic population, about which there is no sound *a priori* information on “where” and “when” potential respondents could be found, which is essential to define sampling frames to use classic methods [33].

DUI is considered a serious public health problem in Brazil. The availability of AO in Brazil as well as in Porto Alegre is considered high. For these reasons, we conducted a study to estimate the prevalence of DUI overall and by sex and broad age group using a population-based sample of individuals who consumed alcohol in AOs in the city of Porto Alegre, Brazil. Sex and age are two of the risk factors often associated with DUI. We found no studies in the literature addressing these questions with a probabilistic, representative sample of this mobile population. Therefore the present paper, besides providing and discussing the prevalence estimates, also describes the procedures adopted for the selection, interviewing and weighting of the sample of individuals attending AOs.

## Methods

### Survey Population

The survey population corresponds to individuals aged 18 years or more (i.e. those with legal age to drive as defined by the Brazilian legislation), who live in Porto Alegre, who have been driving cars or other motor vehicles in the last 12 months and who have drunk on the premises of an AO during the survey reference period. The designation AO is used here to represent bars, nightclubs, restaurants, pubs, gas stations with an attached convenience store, etc. where people buy and consume alcoholic beverages on the premises. This designation does not include outlets where people can buy alcoholic beverages only for taking away but not to consume on the premises. Therefore our study excludes supermarkets, minimarkets, liquor stores, etc. It also excludes shopping malls where many different stores, such as fast food franchises and restaurants cluster in a single area, where tables and chairs are shared. In these “food squares” it is not possible to disentangle the clients of the different facilities without incurring in complex operational and ethical problems.

Most people go to different AOs over time, sometimes visiting more than one in a single day, depending on the period of the day as well as seasonal variations, making the adscription of a given set of individuals to a given AO a highly unlikely assumption. This population must therefore be considered as a mobile population, attending different AOs in the context of different timeframes and locations.

### Ethics Statement

All participants provided verbal informed consent. Considering the environment in which data collection was performed and the possible legal implications, non-identification of the subjects was considered the least invasive form, with the least effect on voluntariness- and written consent was not obtained. However, the entire process of consent was preserved, and the researchers had the duty of informing about the procedures, risks, benefits and rights involved in the study [45], [46]. The subjects received an information letter at the time of their enrollment, with those and contact information. All the procedures were approved by the Ethics Review Board in charge of evaluating and approving the study (HCPA IRB GPPG 06-012). Participants who had a positive BAC and informed they would drive were advised to let a sober peer to drive on their behalf. Alternative transportation (taxi) was also offered through a pre-established agreement with a local taxi company. Following an agreement between the research team and the police, one patrol was fully available in the context of violent neighborhoods to preserve the safety of the research team. This patrol could be easily reached by mobile phones, which all members of the research team had.

### Sample Design

The sample design used was a stratified three-stage cluster sampling. In the first stage, census enumeration areas (CEA) were the primary sampling units. In the second stage, combinations of outlets and shifts (COS) defined the secondary sampling units, and, in the third stage, drivers who drank at the selected COS constitute the tertiary sampling units. By shifts we mean periods of time when the AOs are open and which we considered for sampling and control of data collection operations. For example, a certain outlet might operate three shifts in a day, starting at 12∶00 to 15∶00, then from 15∶00 to 21∶00 and from 21∶00 to 03∶00.

**Figure 1:**
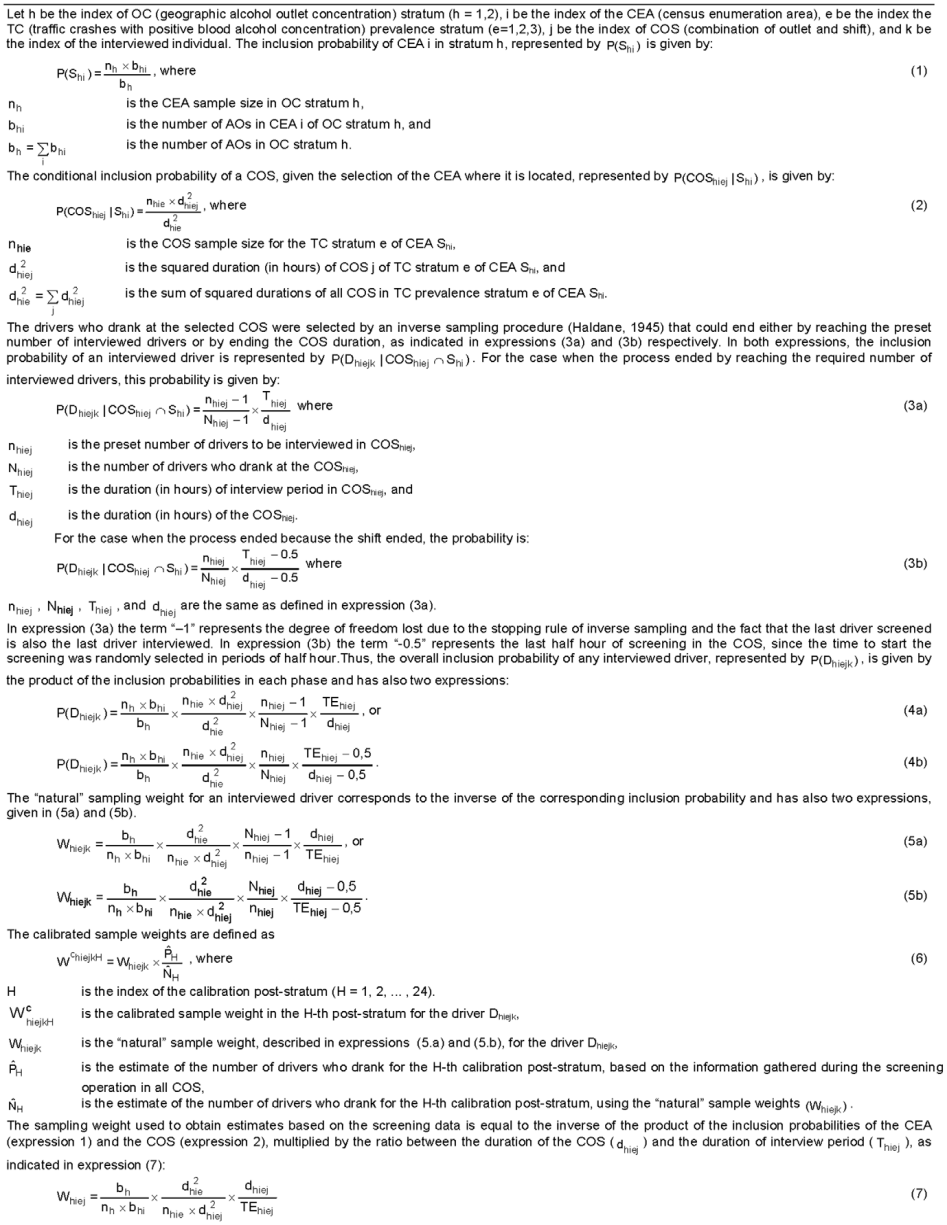
Probability sampling strategy.

The CEAs were first stratified according to the density of AOs in their neighborhood (two geographic strata), and then within each stratum selection was carried out with probability proportional to the number of AOs in each CEA. The COS were stratified (three strata) by prevalence of traffic crashes (TC) with positive blood alcohol concentration, and selection was carried out using probability proportional to size (PPS) where size was their squared duration in hours. In the last sampling stage the drivers were stratified according to their intention to drive after drinking, and then selected using the inverse sampling procedure proposed by Haldane [34] - see Figure 1.

#### CEA stratification

CEAs could be individually classified according to the number of AO in each one. However, to better evaluate the association between the availability of outlets and alcohol consuming patterns, we defined strata using “geographic affinity areas”, i.e. contiguous areas with either high or low density of AO. To define such affinity areas, our research group carried out an exploratory study, using the city’s 2008 commercial licenses database as data source. This database was provided by the Porto Alegre Secretary for Industry and Commerce, and Kernel maps depicting “hot” areas (high density of AOs) were then used to stratify Porto Alegre into areas with high or low concentration of AOs, the latter corresponding to the vast majority of residential areas far from the hot spots [35].

#### Definition of shifts and stratification

Shifts were generally defined using patterns of time of day of TC observed in data obtained by a cross-sectional study described elsewhere [36]. All non-fatal TC victims, who had suffered TC and attended the two Porto Alegre emergency rooms during the 45 days of data collection, answered a structured interview, and were then breathalysed. Frequencies of alcohol related TC were tabulated according to the weekday and 3-hour period of the day, such as 0-3am, 3am–6am, etc. initially aiming to define four “six hour” shifts. The first ( = 1.59) and third ( = 4.76) quartiles of the alcohol–related TC distribution were used as cut-off points to define the three strata: (1) low prevalence (≤1.59; not highlighted in Table 1); (2) intermediate prevalence (>1.59 and ≤4.76; with italics in Table 1); and (3) high prevalence (>4.76; with bold in Table 1).

**Table 1:** Prevalence of alcohol related traffic crashes (ARTC) by shifts (time and day of week), in TC victims attended in emergency rooms of Porto Alegre, 2008.

Day of week	Time			
	03∶00∶01 to 09∶00∶00	09∶00∶01 to 15∶00∶00	15∶00∶01 to 21∶00∶00	21∶00∶01 to 03∶00∶00(+d1)
Sunday	*3.17*	**7.94**	**11.11**	*4.76*
Monday	*3.17*	1.59	**7.94**	*4.76*
Tuesday	0.00	0.00	*4.76*	*4.76*
Wednesday	1.59	0.00	*3.17*	*3.17*
Thursday	1.59	1.59	0.00	*3.17*
Friday	1.59	1.59	0.00	**6.35**
Saturday	**6.35**	0.00	*3.17*	**12.70**

ARTC prevalence strata used in the combination of outlet and shift (COS) stratification: usual font indicates low prevalence of ARTC; *italycs* indicate Intermediate prevalence**; bold** indicates high prevalence.

In order to prepare the frame for the COS selection, an exhaustive enumeration of the AOs in each selected CEA was performed. The opening hours of each AO in each weekday were then recorded. Minor adjustments of the generic shifts defined *a priori* were needed in order to avoid too small shifts (less than one hour) and to adapt the shifts to AOs open exclusively for lunch or dinner. In the latter case, adjustments were up to plus or minus 2 hours. In the end, shift durations varied between 2 and 8 hs, with a mean of 4.8 hours and a mode of 6 hours.

Approximately 11% of the shifts defined were found to be 2 h shifts, a probably insufficient time frame for the purpose of collecting as many interviews as defined by the sampling strategy. In order to make the inclusion of such short shifts less likely, we squared the duration of the shift, in hours, to use as size measures for the PPS sampling of COS.

#### Screening and driver selection

Drivers were selected using inverse sampling: a procedure assessing in a sequence the clients leaving the COS, that would end either by reaching the preset number of drivers to be interviewed at the COS or by reaching the end of the COS duration.

Considering both previous data available in the literature, as well as the putative impact of the recent (2008) federal Law (11.705/08), which imposes heavy sanctions and fines for DUI, we estimated the current prevalence of DUI as 20–25% [37], [38]. Considering that the main focus of the survey was the assessment of those intending to drive under the influence of alcohol, we decided to: a) interview all drivers who had been drinking and intended to drive (DUI drivers), and b) interview one in every four drivers who had been drinking but stated they would not drive (non-DUI drivers).

For this purpose, it was necessary to select, for each non-DUI driver, in every COS, a random start with equal probability between numbers 1 to 4 in order to assign the first interview, and the time of beginning interview in COS using equiprobability between 0 and 60 minutes, adjusted to the nearest half hour.

Two trained interviewers (who were Health or Psychology area students or recent graduates) approached the adults leaving the outlets in the very moment the individuals left. In order to help them in selecting the drivers, a screening data sheet was prepared for each selected COS, containing the identification of the COS, the time when the screening should start, the number of the first non-DUI driver to be interviewed, and information to identify the individuals who belonged to the survey population as well as the screening result. In order to avoid double counting, one of the survey’s screening questions asked the interviewee whether he or she had previously taken part in the survey.

#### Sample size

The survey budget was developed to enable conducting 600 complete interviews. Such a sample, if selected by simple random sampling, would allow estimating proportions with a maximum error of 4%, at the 95% confidence level. As with any survey, we anticipated that sample losses might occur due to various factors, such as unavailability of eligible survey units in certain COS (e.g. daytime shifts when few people drink alcohol beverages, driver’s refusal or incapacity to participate, etc). For this reason, assuming an expected 25% loss of selected sample units, we decided to use a total sample size of 800, which was allocated proportionally to the number of AOs in each geographical AO concentration stratum.

In order to assign the sample size for the CEA and COS samples, the required number of interviews in each stratum was considered. Table 2 provides the target sample sizes by stratum.

**Table 2:** Combination of outlet and shift (COS) and driver sample sizes assigned to each sample stratum.

COS Stratum	Census Enumeration Areas (CEA) Strata			
	Low concentration of alcohol outlets		High concentration of alcohol outlets	
	Number of COS	Number of drivers	Number of COS	Number of drivers
Low ARTC prevalence	2	2	2	2
Medium ARTC prevalence	2	2	3	2
High ARTC prevalence	2	2	3	4

ARTC means alcohol related traffic crashes.

These definitions lead to an overall adjusted sample size of 806 individuals. The number of sampled COS was 334, and the number of sampled CEAs was 48.

#### Sample weighting and calibration of the sample weights

For each driver, the “natural” sampling weight corresponds to the inverse of the product of the inclusion probabilities in each stage, as depicted in Figure 1. For data collected in the screening operation, the sampling weight must consider the inclusion probabilities of the CEA and the COS, as well as the duration of the interview period in relation to the duration of the COS (Figure 1).

Unplanned events in the fieldwork required some flexibility from the interviewers and local supervisor, and the adoption of some ad hoc alternatives. This happened when a selected non-DUI driver refused to be interviewed. As a consequence, the sample weights had to be calibrated to recover the proportion of DUI and non-DUI drivers observed in the screening operation. A post-stratification estimator was used for this purpose.

Geographic AO concentration strata, sex, three age groups, 18–29y, 30–44y, and 45+y [39], and the decision of driving or not after drinking were used to define the 24 post-strata used for weight calibration. The population totals in each post-stratum presented in Table 3 were obtained by multiplying the proportions of DUI and non-DUI drivers of each post-stratum (estimated by using the screening data) to the estimate (151,573) of the population total (eligible drivers who drank at an AO during the survey period) – calculated by using the sample weight of each driver presented in expressions 5a and 5b, in Figure 1.

**Table 3:** Estimated counts in post-strata used for sample weight calibration.

Sex and age groups	High outlet concentration area		Low outlet concentration area	
	Not going to drive	Going to drive	Not going to drive	Going to drive
Men				
18–29y	4,105	3,078	8,693	19,349
30–44y	4,078	5,276	12,818	18,476
45y or +	3,353	3,012	10,302	22,698
Women				
18–29y	3,159	1,444	4,132	4,658
30–44y	2,267	2,004	9,135	4,356
45y or +	1,499	994	2,687	0

### Interviews

Selected drivers answered a structured interview collected by Personal Data Assistants (PDAs) linked to an online database. Alcohol abuse and/or dependence were assessed by “The Alcohol Use Disorders Identification Test” (AUDIT) [40], [41]. Risk perceptions and behaviors associated to DUI were evaluated by a six items scale, as originally proposed by the Global Road Safety Partnership [42]. Four individual questions were added to the original scale, asking about: i) the number of previous accidents, ii) the number of times the interviewee had been stopped for random breath test; iii) the respondent’s opinion about the Law 11.705/08 (i.e. the legislation regulating driving under influence in Brazil, passed as a federal legislation in 2008), iv) and whether the respondent had modified driving behaviors after the implementation of Law 11.750/08.

BAC was estimated using a calibrated breathalyzer (model ALCO-SENSOR IVTM, Intoximeters Inc, Devon, UK), with a correlation coefficient (r) ranging from 0.86 to 0.97 [43], [44]. Positive BAC was considered any different from zero measure, since this is the allowed BAC to drive in Brazil after implementation of the Federal Law 11.705/08.

The screening tests for marijuana, cocaine, benzodiazepines and ecstasy was performed with saliva tests, collected with the help of an oral fluid collection device (QuantisalTM, Immunalysis Corporation, Pomona, CA, USA) and analyzed by an ELISA assay. The sample collection procedures were measured with the help of a stopwatch and results were keyed in the PDAs.

## Results

Table 4 presents estimated and actual sample sizes in each phase and summarizes the strata as defined by the sampling method and as actually used in the field work, as well as the underlying reasons the screening process was discontinued in each specific situation. The differences regarding the estimated and actual number of COS were due to: 1) In ten cases, it was not possible to select the COS (due to their absence in the updated register) and/or to screen the potential interviewees (the selected outlet was actually closed), and 2) In the remaining five cases, the screening process took place, but it was not possible to find a single person who had been drinking. No further weighting was applied to such events. In the vast majority of cases, the *screening* process was discontinued after reaching the number of interviews defined a priori (Table 4).

**Table 4:** Selected and final sample sizes in each selection phase, and COS screening results, by geographic alcohol outlet (AO) concentration stratum.

Sample sizes and COS screening results	Total	Geographic Strata	
		High AO concentration area	Low AO concentration area
**Selected sample size**			
CEA	48	23	25
COS	334	184	150
Drivers	806	506	300
**Final sample size**			
CEA	48	23	25
COS	319	174	145
Drivers	683	443	240
**COS screening results**			
Reached interview preset number	222	129	93
Shift duration ended with interviews	97	45	52
Shift duration ended without interviews	5	2	3
Impossible selection or data collection	10	8	2

CEA means census enumeration area.

COS means combination of outlet and shift.

Table 5 summarizes the results of the screening procedures actually implemented, according to the assumptions and categories used in the study.

**Table 5:** Screening results.

Screening results	Number of individuals						
	Approached	Who lives in Porto Alegre	Who were not previously interviewed	Who were driver	Who drank in the outlet	Who drank and was going to drive	Who drunk and was not going to drive
Total	3,118	2,584	2,562	2,022	1,069	544	525
Driver interviewed	683	683	683	683	683	503	180
Driver not selected (non-DUI driver)	345	345	345	345	345	0	345
Driver that refused	41	41	41	41	41	41	0
Non-eligible person who refused	53	22	19	2	0	0	0
Person unable to respond to interview	3	3	3	0	0	0	0
Person does not drink or does not drive (non-eligible)	1,436	1,436	1,436	951	0	0	0
Person does not live in Porto Alegre, has already been interviewed or age <18 years	557	54	35	0	0	0	0

Table 6 presents the proportions of DUI drivers, based on the information gathered in the screening operation and estimated by using the sample weight presented in expression (7) of Figure 1, as well as the estimates of drivers who drank in the outlets by using the calibrated sample weights. Corresponding standard errors are also presented to enable assessment of the precision of these estimates.

**Table 6:** Estimates of drivers who drank at AO and prevalence of DUI in Porto Alegre, 2009, by sex and by age groups.

Sex and age groups	Number of drivers who drank at an AO (in thousands)		Prevalence of DUI	
	Drivers	SE	%	SE
All	151.6	22.3	56.3	3.5
Sex				
Men	115.2	19.9	62.4	3.8
Women	36.3	6.6	37.0	6.1
Age				
18–29y	48.6	13.1	58.7	6.1
30–44y	58.4	11.8	51.6	4.7
45y or +	44.5	8.8	59.9	7.0

SE is the standard error.

## Discussion

We emphasize that the aim of the study was to estimate DUI prevalence specifically among drivers who drank at AOs. Even though the 2010 Population Census indicated that the city of Porto Alegre has an adult population (18y or +) of 1,083,600 individuals, no one knows how many are drivers (note that it is not uncommon that individuals drive without a driver license, and drivers with a valid license may not drive), and how many drivers are actually drinking on AO at any specific period of time. We have shown that the target population of our study is mobile, and there are no standard sampling frames which could be used to sample from it. These facts limit the possibilities in terms of potential sampling designs and methods for our survey.

Sampling from mobile, elusive and hard to reach populations [48] experienced recent development and discussion. We initially considered several potential alternative designs. Snowball sampling [24] and Respondent Driven Sampling (RDS) [25] are special cases of network sampling designs, where members of the target population are approached and asked to provide lists of their contacts with other population members. Such lists of contacts are then used to sample population members to be approached for the survey at hand. These methods could in principle be used to locate drivers who drink in AOs. Since our survey measurement process required breathalizing and taking saliva samples of the drivers just after drinking at an AO, both methods would not be suitable to locate such drivers at the right time and place.

We also considered standard Time Location Sampling [29], [30], [31] designs. However, this approach suffers from the fact that at the last stage of sampling there are no grounds on which to compute sample inclusion probabilities. Therefore such methods are not able to provide estimates of the population size, and neither estimates of the precision (standard error) of the prevalence and other parameter estimates.

Our approach, which combined the use of venues and time periods to define a sampling frame (a well known advantage of TLS) used up to the sampling of AO and shift combinations, with inverse sampling in the last sampling stage, enabled us to obtain a strict probability (representative) sample of this mobile population. The design used in the present study enabled calculation of the inclusion probability for each one of the sampling stages, and as a consequence, we could not only estimate the overall target population size, but also prevalence and other parameters with corresponding standard errors.

The combined use of different methods such as stratification and inverse sampling enabled a good trade-off between resource and time allocation, while preserving the ability to generalize the findings. In the effort to obtain the best sample given the available resources, the *a priori* assessment of a high proportion of losses during fieldwork was pivotal for the posterior success of the study. Losses were due to a combination of reasons: impossibility to survey a given COS when valid outlets in the updated frame were actually closed; frustrated attempts to complete the predefined interview number for a given shift, due to refusals or to the small number of people who have drunk in a given facility selling both alcoholic beverage and meals (for instance, during lunch time) or due to the fact that people did not actually leave the facility for the whole duration of a given shift. In our case, actual losses were inferior to the expected number of losses, indicating that we succeeded in our data collection strategy.

Calibration is often used to ensure that survey-based estimates match known population counts or totals by strata. For the sake of calibration, one usually profits from triangulation with data provided by additional sources. In the present study, calibration was based on matching to estimates derived from the survey’s own screening operation (a first phase sample). Estimators such as this one belong to the family of post-stratification estimators, as discussed by Särndal, Swensson & Wretman [47]. Such estimators have properties similar to those estimators, which consider as auxiliary population information known stratum totals or counts. Both estimators will be approximately unbiased when the probability of response remains approximately constant in each one of the post-strata used for calibration. This condition was actually observed in the present study. However, calibration procedures based on estimated stratum counts or totals usually have larger variance and require a more complex calculation than calibration based on known population stratum totals and counts. Variance estimation for such calibration estimators require taking into account the calibration residuals [58]. Despite these caveats, the calibrated estimators used in the survey provided reasonably precise estimates, as can be seen by the standard errors presented on Table 6, and the necessary calculations can be carried out using the “survey library” in R language [58].

We acknowledge here that the proposed sampling strategy may be viewed as a limited strategy due to the *a priori* assumption that venues are necessarily associated with physical locations/facilities (such as AO), which permit the elaboration of an exhaustive list of locations to be further selected in the subsequent steps (selection frame). In this sense, such a strategy could not be used for other scenes and settings, such as parties, raves or locations where people consume illicit drugs and alcohol in hidden locations due to the impossibility of defining physical structures with a stable location such as the AOs. However our team has successfully adapted the sampling design presented here for the National Survey on Crack-Cocaine Users, currently being implemented by the authors for the Brazilian National Secretariat for Drug and Alcohol Policies.

Note also that inverse sampling assumes that potential respondents should be approached following a random sequence; in our case this was the order in which people left the AOs. On the other hand, under the conditions of data collection for the empirical study, we had no better way to control the sampling of potential drivers after they had been drinking at the sampled AOs.

The fact that the target population for our study was drivers who had been drinking at an AO implies that it is not possible to express the DUI prevalence found (56.3%) with respect to total adult population of the City of Porto Alegre. Nevertheless, the estimation of the prevalence of alcohol harms and risks for those drinking on AOs is a theme of growing importance in the international scenario, possibly because these places may become targets of public policy (such as limiting opening hours, for example [9]). The clients of different AOs may be subjected to different harms and risks, such as accidents (as discussed here), the misuse of multiple drugs, including alcohol and illicit drugs, as well as risky sex behaviors [49], [50]. All such harms and risks constitute public health concerns, but have been basically assessed using non-probabilistic sampling methods, which may compromise the accuracy of estimates and the ability to generalize the findings [50], [51].

Overall, the prevalence of “intending to drive after alcohol consumption” was found to be much higher (56.3%) than previously anticipated. It seems to remain high despite the adoption of a much more strict law since 2008. Such disquieting high prevalence of a well-known risk behavior and major public health problem, in spite of the passing (but, much probably, not an optimal enforcement) of a more restrictive legislation, is pivotal in terms of a better definition of public policies aiming at minimizing the adverse consequences of alcohol misuse. Some international studies have already indicated that impaired driving prevalence is high among individuals who attend drinking environments, like bars and clubs. Furr-Holden et al., for example, in 2006, have found a prevalence of DUI (either under influence of alcohol alone or alcohol plus another substance) around 50% among attendees of electronic music events [51]–[53]. However, considering the use of different methodologies, as well as the unique combination in Brazil of the recent implementation of a new zero-tolerance legislation – yet to be fully enforced – and the absence of restrictions on alcohol selling, make the comparison of our findings with the international literature a challenge yet to be properly addressed.

The findings of the screening process corroborate data previously discussed by the international literature. For instance, the clientele of the AOs was predominantly comprised of men, a common finding usually associated with the higher number of serious and fatal car accidents among men, compared to women [54], [55]. However, differently from international studies, but in harmony with previous Brazilian studies [36], [37], [56], no significant difference was found between the drinking and driving behavior between individuals aged 18–29 and 30–44, probably due to the lower availability of cars for younger drivers in Brazil, as well as to different alcohol usage patterns in the Brazilian population – where binge drinking prevalence is high until 45 years old [57].

From a methodological point of view, such high prevalence may suggest that, at least in Brazil, future studies may not need to select just a ¼ proportion of non-DUI drives, which may help to simplify and make studies cheaper.

Given that the data were obtained using a complex sample design, involving stratified multistage sampling with unequal probabilities of selection, as well as the calibration for the weighting of responding drivers, we strongly recommend that all data analysis carried out take into account the weights and other structural design information available. This task is made easier these days by the relatively wide availability of specialized software, which can perform survey estimation and data analysis using appropriate methods, that take into account that the data were collected by complex sample designs.

Despite these limitations, the current strategy can be viewed as a step forward in the efforts to better estimate hard-to-reach, mobile populations and an essential tool for the formulation and monitoring of public policies aiming at reducing accidents related to alcohol, as well as other alcohol-related harms and injuries. The estimates obtained in the study are valid for the survey population of adult drivers in Porto Alegre who drank at an AO during the survey period, since our strategy used verifiable methods, which can be independently replicated by others. This is clearly a step forward regarding improving validity of studies of such mobile and hard to reach populations.
